# A rare combination of Dermoid cyst and Cystadenoma: Are collision tumors in the ovary a real entity?

**DOI:** 10.4322/acr.2021.249

**Published:** 2021-03-12

**Authors:** Tushar Kalonia, Neha Kumari, Akanksha Malik, Arvind Kumar, Anupama Bahadur, Sanjeev Kishore

**Affiliations:** 1 All India Institute of Medical Sciences, Rishikesh,Tushar Kalonia, Deapartment of Pathology and Laboratory Medicine. Rishikesh, Deharadun, Uttarakhand, India; 2 All India Institute of Medical Sciences, Rishikesh, Tushar Kalonia, Department of Gynecology and Obstetrics. Rishikesh, Deharadun, Uttarakhand, India

**Keywords:** Cystadenoma, Mucinous, Ovary, Teratoma

## Abstract

Collision tumors have been reported in various organs like the gastrointestinal tract, lung, skin, adrenals, central nervous system, lymph nodes, uterus, but are rarely seen in the ovary. Collision tumors are two histologically distinct neoplasms in the same organ without any intermixture between them. Here we present a case of a collision tumor of the ovary represented by a mucinous cystadenoma and teratoma. It is imperative for a surgical pathologist to correctly diagnose the collision tumor components and differentiate them from mixed tumors as it will dictate the appropriate treatment based on the individual biological aggressiveness of each component.

## INTRODUCTION

Collision tumor refers to the simultaneous coexistence of two distinct tumors in the same tissue without any transition zone or mixing interface. Collision tumors have been described in various body organs, e.g., liver, bone, kidney, brain, lung. However, collision tumors in the ovary are rarely seen.^1^ Case reports of collision tumors in the ovary were reported depicting the admixture of granulosa and cystadenocarcinoma, granulosa and teratoma, serous adenocarcinoma, and steroid cell tumor.^2^^-^^4^

The cystadenomas account for 30% of the ovarian tumors, and the mature cystic teratomas comprise 10-20% of the ovarian tumors.^5^ However, together only 2-10% of teratomas are associated with mucinous cystadenomas.^6^ Mature cystic teratoma co-existing with a mucinous cystadenocarcinoma is infrequently encountered, with only a handful of cases reported to date.

Here we report a collision tumor of the ovary with the simultaneous association of dermoid cyst and benign mucinous cystadenoma.

## CASE REPORT

**Figure 1:**
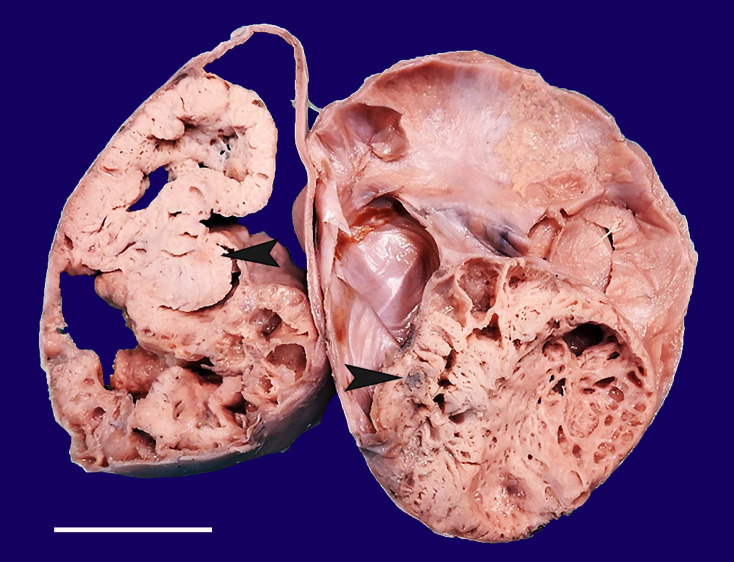
Gross view of the cut surface of the tumor with cystic and greyish-white solid area(arrowheads) that represent teratomatous component in microscopy.

A 36-year-old female was referred to the Gynecology Department complaining of dysmenorrhea for the past 3 months associated with intermenstrual spotting. The abdominal examination revealed a freely mobile mass in the right lower abdominal quadrant. The pelvic CT scan revealed cystic lesions on bilateral adnexa, the left side with 7.4x6.2x6.1 cm, and the right side with 14.5x17.4x18.5 cm.Thus, the bilateral adnexal masses were excised along with hysterectomy. The cut surface of bilateral ovaries shows solid and cystic areas with focal papillaroid areas along with an occasional greyish brown, firm area, which revealed a teratomatous component (Figure 1).

**Figure 2:**
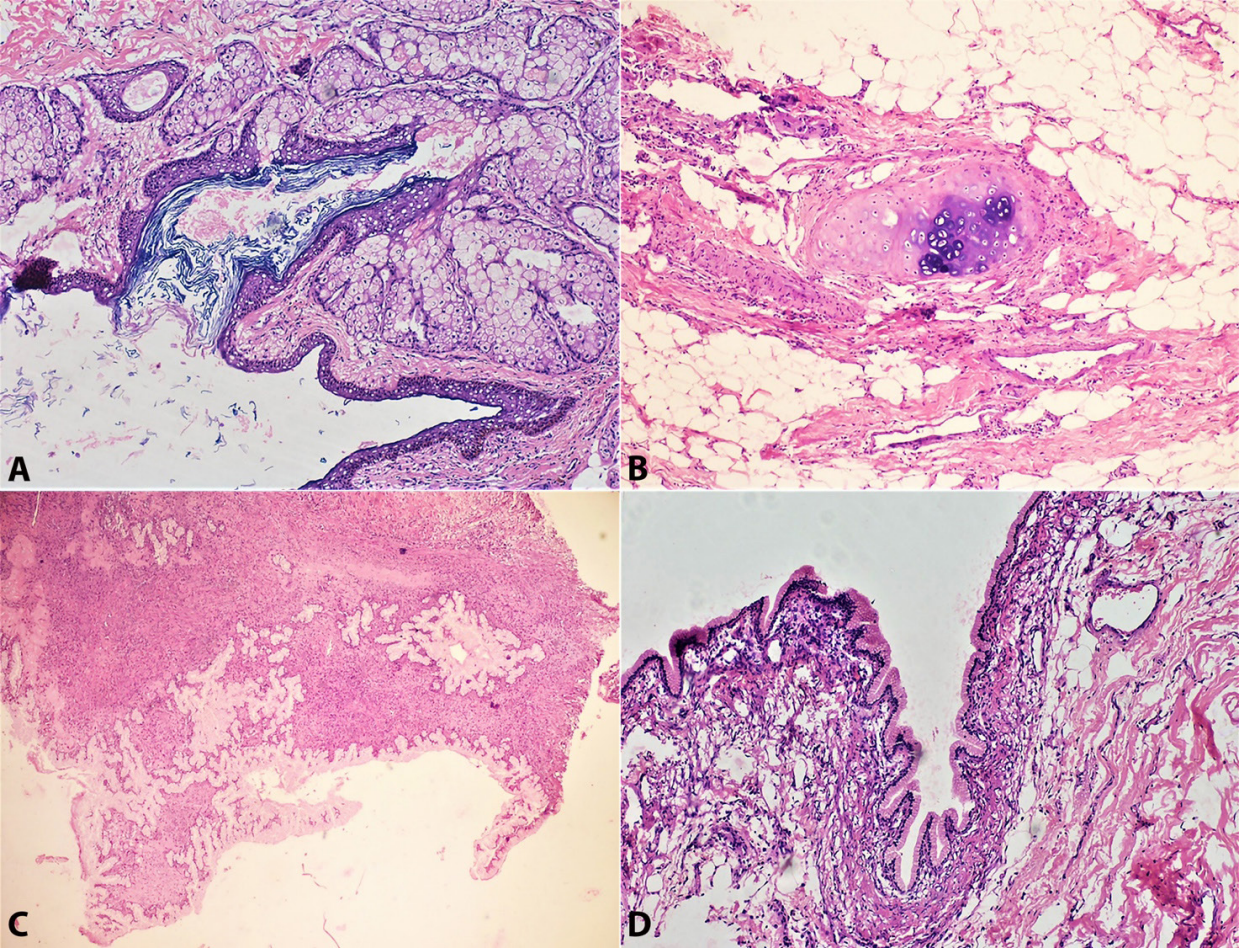
Photomicrographs of the tumor. **A –** Section is lined by keratinous stratified squamous epithelium. The sub epithelium shows lobules of glands (H&E,100X); **B –** The section shows a cartilage formation (H&E,200X); **C –** Part of the cyst cavity is lined by benign columnar epithelium with moderate amount of mild eosinophilic cytoplasm. Tangential cut of the glands are also noted (Frozen Section,100X); **D –** The section is lined by columnar glandular epithelium having basally located nucleus with bland nuclear feature and moderate amount of eosinophilic cytoplasm(H&E,100X).

The Paraffin-embedded samples from left adnexa stained with H&E stain showed solid cystic ovarian parenchyma partially lined by stratified squamous epithelium along with the presence of pilosebaceous units, cystic spaces filled with keratinous debris with pigment-laden macrophages in its wall. A diagnosis of mature cystic teratoma was rendered on the left adnexa. Right adnexa showed ovarian stroma and many cysts like spaces lined by simple non-stratified columnar cells with abundant intracellular mucin and distinct areas of cartilaginous differentiation, pilo sebaceous units, and keratinous debris separated by ovarian parenchyma. (Figure 2 A to D). Hence a diagnosis of a mature cystic teratoma with mucinous cystadenoma was given However ,intraoperative frozen sections examination was reported as suggestive of mucinous cystadenoma on both sides. (Figure 2C)

## DISCUSSION

The origin of teratomas is still widely disputed. The most accepted theory is that they arise from primordial germ cells.^6^ The diagnosis of a collision tumor is made when normal tissue intervenes between both tumors, and there is no histological admixture at the interface. The occurrence of a transition zone between the two tumors makes it difficult to differentiate between two colliding tumors or a true mixed tumor.^3^^,^^7^

The histogenesis of the ovarian mucinous cystadenoma has not yet been fully clarified. There has been the suggestion of a surface epithelial metaplasia origin or a teratomatous origin. The ultrastructure studies and mucin histochemical studies have supported the surface epithelial metaplasia theory.^8^^,^^9^ The frequent co-existence of mature cystic teratoma and mucinous cystadenoma is the basis for the teratoma theory. Germ cell tumors comprise approximately 30% of the primary ovarian tumors, and of these, 95% are mature cystic teratomas. The most common histological combination of collision tumors in the ovary is the coexistence of teratoma with mucinous tumors.^10^ The main differentiating feature between collision tumor and teratoma with cystadenoma component is that the former presents a distinct collar of normal ovarian parenchyma separating the teratoma and cystadenoma component, while the latter entity shows an intermixture of both the histological components. Various hypothesis has been proposed for the existence of collision tumors in the ovary. The first hypothesis is that the first tumor’s presence causes changes in the tissue microenvironment, making it conducive for the seeding of a metastatic tumor or development of the second tumor. The second theory proposes that the existence of two primary tumors is just a chance occurrence. This theory was supported by Vang et al.,^11^ who showed the ovarian mucinous tumors associated with mature cystic teratomas exhibited morphological and immunophenotypic diversity. The third theory proposes that both the primary coexisting tumors have a common stem cell origin.^12^

The evidence of a clonal relationship could be provided by additional molecular analyses demonstrating shared genetic changes in the mucinous tumor and adjacent conventional teratomatous elements. Thus, supporting the theory of a teratomatous origin for these mucinous tumors.

In a study by Fuji et al.^13^ the homozygous genetic patterns similar to those of the teratomatous components were shown in mucinous tumors arising together with mature cystic teratomas, and it was suggested that these mucinous tumors developed from pre-existing mature cystic teratomas.

Although not all mucinous tumors are of germ-cell origin, mucinous elements in dermoid cysts may be of teratomatous origin as they are more likely to be intestinal rather than Mullerian in differentiation.^14^

Another study published stated that molecular findings which are significant allelic imbalance for microsatellite markers, indicating homozygosity rather than heterozygosity for chromosomal polymorphisms in some ovarian mucinous cystadenomas associated with mature cystic teratomas, were consistent with a germ cell origin.^15^ Okada et al.^16^ demonstrated a possible association between dermoid cyst and multiseptated cyst. Multiseptated cyst contains fatty tissue foci. It was also stated that recognizing of the potential for the coexistence of these two neoplasms in the same ovary is essential to make a correct diagnosis.

## CONCLUSION

Collision tumors involving ovaries are rare. The surgeon should establish good perioperative communication with the pathologists and whenever possible, a specialized gynecology pathologist is advisable since a thorough grossing and recognition of close differential diagnosis lead the correct diagnosis.
